# Anecdota Sydenhamiana: Medical Notes and Observations of Thomas Sydenham, M. D., Hitherto Unpublished

**Published:** 1845-10

**Authors:** 


*Anecdota Sydenhamiana: Medical Notes and Observations of Thomas Sydenham, M. D., hitherto unpublished* 24mo. pp. 80. Oxford: 1845.

The Editor of this small *brochure* has entered upon a career of eminent usefulness. Estimable, learned and pious, he is now engaged in writing the lives of many of the older physicians in the excellent Biography in course of publication by the Society for the Diffusion of Useful Knowledge, and was selected by the

Sydenham Society—that most useful association—to edit the works of Sydenham, and to revise the Latin original, a task, which he executed in a manner that satisfied every one. He is now—as mentioned in another part of this number—issuing a prospectus, according to which he proposes "with God's assistance, to publish in a series the lives of those physicians who have been most eminent for their piety, in whatever age and country they may have lived"—the profits, if any, accruing from these publications, to be given eventually to some medical charity.

The *Anecdota Sydenhamiana* are taken from a MS. in the Bodleian Library at Oxford. The name of the writer is not mentioned, nor is anything known of the history of the MS., except that it once belonged to Dr. Richard Rawlinson, and forms part of the collection of MSS. bequeathed by him to the University of Oxford about the middle of the last century.

"At the beginning of the volume (of which about two-thirds have been torn away) are these:—'Extracts of Sydenham's Physick Books, and some good letters on various subjects.' This is the whole of the evidence respecting the genuineness of the following Anecdota; and perhaps, if there were nothing more to say in their favour, it might be doubted how far the editor was justified in giving them to the world under the sanction of the name of Sydenham: the *internal* evidence, however, is much more conclusive, and indeed to his own mind perfectly satisfactory. The writer professes to have been acquainted with Sydenham himself, and to have originally written the following notes, partly from his dictation, in the years 1682—83, and partly from some of his MSS., written chiefly in 1670. These notes he appears to have revised, and written out correctly in their present form *after* 1685, (as he refers to the edition of Sydenham's works pnblished in that year, and if the editor's conjecture at page 69 be correct) before 1692—as that is the date of the first edition of the *Processus Integri.* The undesigned coincidences, however, between these Anecdota and Sydenham's acknowledged works, which must strike every one who is at all familiar with his writings, constitute the most convincing proof of their genuineness. These are too numerous to be pointed out, but references have been given to the chapters of the *Processus Integri,* by means of which the reader, if he possesses the edition of Sydenham's works published by the Sydenham Society, may easily find out for himself other parallel passages." p. v.

We have no doubt of the authenticity of the *Anecdota*, and consider that every one who possesses the other works of the illustrious English physician should become possessed of this. Dr. Greenhill has appended an index of the drugs, wherein the words are printed at full length, and accompanied by the scientific and the common English names. The spelling of the MS. has been followed implicitly, and in general the punctuation likewise.

